# Current status and influencing factors of the second victim experience and support of operating room nurses

**DOI:** 10.3389/frhs.2025.1527983

**Published:** 2025-05-15

**Authors:** Yan Zheng, Yinghao Zhou, Junhua Fu, Dan Zhou, Liang Lu, Xiuzhi Yang, Yali Li, Lin Zhao

**Affiliations:** ^1^ Operatina Room, The Affiliated Hospital of Qingdao University, Qingdao, China; ^2^ Spinal Joint Surgical Care Unit, The Affiliated Hospital of Qingdao University, Qingdao, China; ^3^ Nursing Department, The Affiliated Hospital of Qingdao University, Qingdao, China

**Keywords:** operating room, nurse, second victim, career resilience, perceived organization support

## Abstract

**Background:**

As the second victim, nurses may suffer severe physical and mental damage. How to reduce the trauma level of operating room nurses after adverse events, enhance career resilience, and stabilize the nursing team has become an urgent concern for operating room nursing managers.

**Objective:**

To investigate the experience of operating room nurses as the Second Victim of adverse events, and analyze the risk factors affecting the psychological status of operating room nurses as the Second Victim.

**Methods:**

From September to October 2023, convenience sampling was used to select 224 operating room nurses from a Class III Grade A hospital in Shandong Province as the research object. The general information questionnaire, Second Victim Experience and Support Scale, Career Resilience Scale and Organizational Support Scale were used to investigate.

**Results:**

The score of second victim experience and support was 94.32 ± 15.54. Correlation analysis showed that the second victim experience and support of operating room nurses were negatively correlated with career resilience (*r* = −0.383, *P* < 0.01) and organizational support (*r* = −0.272, *P* < 0.01). The results of multiple linear regression analysis showed that responsibility in the occurrence of adverse events, career resilience and organizational support were the influencing factors of victim experience and support of Operating Room nurses (*P* < 0.05).

**Conclusions:**

Most of the second victim experience of operating room nurses is at a medium or high level. Responsibility in adverse events, career resilience and organizational support have a significant impact on the second victim experience of operating room nurses. As a management level, crisis support and external help should be provided for the second victim, and a support system based on the hospital or department level should be established to provide professional and psychological support for nurses and enhance the professional identity of nurses in the operating room at work, so as to further improve the quality of nursing in the operating room and promote the construction of clinical nursing services.

## 1 Introduction

With the advancement of surgical techniques, operating room workflows have become increasingly complex and precise. It is difficult to avoid some instances of suboptimal care, such as surgical instrument miscounts, perioperative pressure injuries, and occupational exposures (1). The risk of adverse events in the operating room is often higher, which will bring greater psychological trauma to the operating room nurses than the ward nurses (2). Additionally, their extensive mental and physical exertion and high levels of stress make them more susceptible to intentions to resign from their positions (3). The Second Victim phenomenon refers to the occurrence of an adverse event in a medical environment that may result in two victims. Patients and their families are the first victims and should be given care, while medical staff are considered as the Second victims, and their psychological status also needs to be paid attention to (4). In the field of health care, patient safety has always been a topic of concern for medical staff. In recent years, medical disturbances have occurred frequently, and the physical and mental health of medical workers in adverse medical events has attracted the attention of society and scholars. After an adverse event occurs, both the event itself and the handing of the event will have an impact on patients and medical staff. If the medical institutions cannot provide enough support for medical staff, huge medical risks will be buried (5).

Several factors may contribute to the nurse's experience following an adverse event. Career tenacity is the concrete concept of the combination of resilience and career situation, which is not only the flexible adaptation to the career environment, the persistence and endurance of challenges, but also the rebound or recovery from pressure and setbacks (6). Studies in China have shown that career tenacity enhances employee motivation and occupational stress coping capabilities. Individuals with higher level of career tenacity have stronger inner self-drive and stronger resilience when they suffer adversity at work (7, 8). Therefore, the role of career tenacity in nurses after adverse event deserves attention.

Social Support Theory emphasizes the importance of relationships and networks in providing emotional, informational, and practical assistance. According to the main effect model of social support, support from the outside society or organization can help the development of individuals (9). Studies have shown that nurses may suffer severe physical and mental damage after experiencing adverse events. And the more support they receive, the easier it is to help them return to a positive state (10, 11). Despite the research of the Second Victim experience of nurses, career tenacity, social support, there is no relevant research on the association among their in the operating room nurses in China yet.

This study aims to explore the current situation and influencing factors of the Second Victim experience of operating room nurses, analyze the correlation between the Second Victim experience, career tenacity, and social support. According to the results of this study, intervention measures can be developed to ensure that operating room nurses maintain a positive and stable psychological state after the occurrence of adverse events, and provide a reference for improving the occupational flexibility of operating room nurses and improving the quality of nursing.

## 2 Methods

### 2.1 Participants

From September to October 2023, we conducted a cross-sectional survey among nurses in operating room from three Tertiary Grade A hospitals in Shandong Province using the Questionnaire Star platfrom. These hospitals represent the highest echelon in China's hierarchical hospital accreditation system, as defined by the National Health Commission. These institutions serve as comprehensive medical centers demonstrating excellence across clinical care, medical education, and scientific research (12). The inclusion criteria were: (a) registered nurses who obtained the nurse practice certificate of the People's Republic of China; (b) clinical experience in the operating room was 3 months or more; (c) adverse events occurred in the process of clinical nursing work; (d) informed consent and voluntary participation in this study. The exclusion criteria were: (a) Non-unit nurses, such as refresher nurses, trainee nurses; (b) those who were on vacation or went out for study during the survey period.

The sample size of this study was calculated by 10–20 times of the independent variable, and combined with the sample attrition rate of 20% (13), the sample size was calculated to be 168–336 cases. A total of 224 questionnaires were collected.

### 2.2 Procedure

We collected questionnaires online. After obtaining the support and cooperation of the hospital nursing management staff, the link of the electronic questionnaire was distributed to the head nurses of the operating room. The researcher assisted the head nurses to screen nurses who met the inclusion and exclusion criteria of this study, explained the purpose and significance of the study in detail. The investigated nurses could click the link to fill in the questions directly, and submit them after completing all the questions. There was no limit to the time and place of answering the questions, but each ID was only allowed to answer effectively once. In this study, a total of 235 questionnaires were distributed and 224 valid questionnaires were returned, with an effective response rate of 95.3%.

### 2.3 Ethical considerations

This study was approved by Ethics Committee Medical College of Qingdao University (QDU-HEC-2021102). Detailed information were provided by the researchs to all respondents. Nurses were informed that they were free to refuse to participate or withdraw from participation at any time without penalty. Written informed consent was obtained from each participant after explaining the anonymity and confidentiality of their participation.

### 2.4 Instruments

#### 2.4.1 Sociodemographic characteristics

The researchers were self-compiled according to the purpose and content of the study, including gender, age, working years, employment nature, professional title, average weekly working hours, education background, and adverse events.

#### 2.4.2 The second victim experience and support tool (SVEST)

The original scale was compiled by American scholar Burlison based on literature review and clinical practice (14). The Chinese version of SVEST was translated and revised by Chen Jiajiao (15). The scale included 6 dimensions of psychological distress, physical distress, practice distress, colleague support, management support and family and friends support, with 24 items. The scale was scored by 5-point Likert scale ranging from 1 (strongly disagree) to 5 (strongly agree). The psychological, physical, and practice distress dimensions were positive, and the colleague, management, and family and friend support dimensions were reverse scored. The total score of the scale ranges from 24 to 120, with higher scores indicating greater impact of the patient safety incident on the second victim and less support for the second victim. The total Cronbach's *α* coefficient of the Chinese version of SVEST was 0.862. The Cronbach's *α* coefficient of each dimension was 0.796–0.917.

#### 2.4.3 The career tenacity scale

The scale was compiled by Chinese scholar Zhu Xuemei in 2021 after literature review and expert letter consultation (16). The scale has 32 items, including 6 dimensions of professional emotion, cooperative spirit, adaptability, self-efficacy, career goals and learning growth. It can evaluate the career resilience of nursing staff in China. Likert 5-point scoring method was used in the scale, which was given 1 to 5 points from “very consistent” to “completely inconsistent”. The higher the score, the higher the level of career resilience of nurses. The total Cronbach “*α* coefficient of the scale was 0.956, and the Cronbach” *α* coefficient of each dimension was 0.790–0.888, which showed good reliability and validity.

#### 2.4.4 The organization support scale

Zuo Hongmei developed the scale based on organizational support theory and according to the work situation of clinical nurses (17). The scale was divided into two dimensions of affective support and instrumental support, with a total of 13 items, which was suitable for nurses to evaluate organizational support. The Likert 5-point scale was used, with 1–5 points indicating “strongly disagree” to “strongly agree”. Each item is rated positively on a scale of 13–65, with higher scores indicating higher perceived organizational support of the individual. The Cronbach's *α* coefficient of the scale was 0.92, which showed good reliability and validity.

### 2.5 Statistical analysis

We managed and analyzed all data by SPSS 26.0 software. The general data were described by frequency and percentage, and the scores of nurses' second victim experience, career tenacity and organizational support were described by mean and standard deviation. Pearson correlation analysis was used to test the correlation between the three. The nurses' second victim experience was used as the dependent variable, and the meaningful variables in univariate analysis and correlation analysis were used as independent variables for multiple linear stepwise regression analysis. Statistical significance was specified at *P* < 0.05 for all test.

## 3 Results

### 3.1 Sociodemographic characteristics of the participants

A majority of the 224 respondents completing the sociodemographic survey were female, aged between 26 and 35, worked less than 5 years (Table 1). And the mean score for the nurses' second victim experience, career tenacity and organizational support were 94.32, 118.73 and 48.48 (Table 2).

**Table 1:** Sociodemographic characteristics (*n* = 224).

Variables	Groups	Number	Frequency (%)
Gender	Male	23	10.27
	Female	201	89.73
Age	18∼	62	27.68
	26∼	102	45.54
	36∼	40	17.86
	>45	20	8.93
Number of years spent working	≤5	104	46.43
	6∼10	41	18.30
	11∼15	42	18.75
	16∼20	12	5.36
	>20	25	11.16
Job specification	Authorized strangth	50	22.32
	Contract system	144	64.29
	Human agency	11	4.91
	Others	19	8.48
Professional titles	Nurse	69	30.80
	Senior nurse	63	28.13
	Nurses in charge	73	32.59
	Co-chief nurse and above	19	8.48
Working hours per week	≤40 h	74	33.04
	40 h∼	67	29.91
	50 h∼	76	33.93
	≥60 h	7	3.13
Highest level of education	Junior college and below	35	15.63
	Bachelor	182	81.25
	Master and above	7	3.13
Marital status	Unmarried	85	37.9
	Married	139	62.10
Liability for adverse events	Primary responsibility	17	7.59
	Secondary responsibility	15	6.70
	Non-liability	131	58.48
	Unclear	61	27.23
Management after an adverse event	N0	181	80.80
	Notice of criticism	34	15.18
	Withholding performance	7	3.13
	Transferred from the original post	2	0.89

**Table 2:** Descriptive statistics for Major study variables (*n* = 224).

Items	Mean	SD
Nurses’ second victim experience	94.32	15.54
Career tenacity	118.73	26.51
Organizational support	48.48	10.97

### 3.2 Single-factor analysis of the nurses' second victim experience

Single-factor analysis was conducted with the total scores indicating the nurses' second victim experience for operating nurses as the dependent variable and with sociodemographic characteristics as the independent variables. The results showed that the experience and support degree of the second victim of operating room nurses with different ages, working years, technical titles and different responsibilities in adverse enents differed significantly (*P* < 0.05). And the other demographic variable did not have any statistically significant differences (*P* > 0.05) (Table 3).

**Table 3:** Single-Factor analysis of the occupational stress for emergency nurses (*n* = 224).

Variable	Mean ± SD	*t*/*F*	*P*
Age (years)			
18∼	100.23 ± 14.72	7.726^b^	0.000**
26∼	94.55 ± 15.03		
36∼	89.75 ± 15.27		
>45	84.00 ± 13.76		
Gender			
Female	93.56 ± 15.30	2.194^a^	0.437
Male	101.00 ± 16.42		
Number of years spent working			
≤5	98.20 ± 15.72	4.699^b^	0.001**
6–10	93.39 ± 13.59		
11–15	92.12 ± 13.85		
16–20	91.92 ± 18.09		
>20	84.56 ± 14.88		
Highest level of education			
Junior college and below	98.80 ± 14.96	1.734^b^	0.179
Bachelor	93.50 ± 15.59		
Master and above	93.29 ± 15.53		
Marital status			
Merried	91.73 ± 15.33	3.264^a^	0.886
Unmerried	98.57 ± 15.03		
Professional titles			
Nurse	97.51 ± 15.64	3.792^b^	0.011*
Senior nurse	94.68 ± 15.29		
Nurses in charge	93.60 ± 15.21		
Co-chief nurse and above	84.32 ± 13.81		
Job specification			
Authorized strength	91.58 ± 15.21	2.058^b^	0.107
Contract system	94.03 ± 15.74		
Human agency	99.55 ± 14.62		
Others	100.74 ± 13.92		
Liability for adverse events			
Primary responsibility	105.42 ± 12.92	3.330^b^	0.020*
Secondary responsibility	95.20 ± 12.83		
Non-liability	93.02 ± 15.80		
Unclear	93.80 ± 15.33		
Management after an adverse event			
N0	93.39 ± 15.39	1.857^b^	0.138
Notice of criticism	96.68 ± 16.35		
Withholding performance	102.43 ± 12.79		
Transferred from the original post	110.50 ± 9.19		

^a^ *t*, the test statistic of the Independent-Sample *T* Test.

^b^ *F*, the test statistic of the one-way ANOVA.

* *P* < 0.05. **P < 0.01.

### 3.3 Correlations between nurses' second victim experience and support, career resilience and organizational support

Pearson correlation test was used to study the relationship between the main variables. The results showed that the second victim experience and support of operating room nurses were negatively correlated with career resilience (*r* = −0.383, *P* < 0.01), and the second victim experience and support of operating room nurses were negatively correlated with organizational support (*r* = −0.272, *P* < 0.01). Career resilience of Operating room nurses was positively correlated with organizational support (*r* = 0.392, *P* < 0.01)(Table 4).

**Table 4:** Relationship between variables.

Variables	Nurses’ second victim experience and support	Career resilience	Organizational support
	*r*		
Nurses’ second victim experience and support	1		
Career resilience	−0.383**	1	
Organizational support	−0.272**	0.392**	1

** *P* < 0.01.

### 3.4 Multiple linear regression analysis of nurses' second victim experience and support

Taking the total score of the Second Victim Experience and Support Scale for Nurses as the dependent variable, the statistically significant variables in the general information and the score of the Career Resilience Score and Organizational Support Scale as the independent variables were included in the multiple linear regression analysis. The results of multiple linear regression analysis showed that liability for adverse events, career resilience and organizational support were the influencing factors of the experience and support of victims of operating room nurses (*P* < 0.05). A significant regression equation was found with an *R*^2^ of 0.311(*F* = 8.679, *P* < 0.05) (Table 5).

**Table 5:** Multiple linear regression analysis of predictor associated with nurses’ second victim experience and support (*n* = 224).

Independent variable	*B*	SE	*β*	*t*	*P*
Constant	133.881	5.146		26.015	<0.001
Career resilience	−0.187	0.037	−0.319	−5.111	<0.001
Organizational support	−0.257	0.089	−0.182	−2.902	0.004
Liability for adverse events	11.829	3.422	0.202	3.457	0.001

*R*^2^ = 0.311, *F* = 8.679, *P* < 0.05.

## 4 Discussion

### 4.1 Current situation analysis of the Second Victim experience of operating room nurses

As demonstrated by the findings, the score of second victim experience and support was 94.32 ± 15.54. Among them, the score of psychological distress is the highest, the average score of items was 4.08 ± 0.93. This indicates that when adverse events occur, operating room nurses are highly affected as Second Victims and are prone to psychological problems such as guilt and anxiety (18).

However, this study found that the scores of all dimensions of the experience of nurses in operating room as the Second Victim are slightly higher than those in intensive care unit, psychiatric department and other departments (19–21). The possible reason is that operating room is an important place for surgical treatment, disease diagnosis and rescue of critically ill patients, and intraoperative cooperation has high requirements for professionalism and rigor. Nursing work in the operating room is directly related to the life safety of patients, so adverse events in the operation often have more serious consequences than those in other departments. As the Second Victim, nurses, in addition to facing family members' doubts and dealing with the follow-up of the incident, also have to report, discuss and analyze adverse events step by step, which makes nurses constantly doubt their clinical ability (22). As the Second Victim, the operating room nurses will also have a deep impact.

### 4.2 Influencing factors of the operating room nurses' second victim experience

The results of multiple liner regression anaysis show that liability for adverse events, career resilience and organizational support were the influencing factors of the experience and support of victims of operating room nurses.

Nurses who were primarily responsible for adverse events were more affected. Some studies have found that 66.0% of medical staff have negative emotions such as tension and anxiety after the occurrence of adverse events, and have a sense of distrust in their ability to perform their work (23). If the Second Victim of nurses bears the main responsibility in the adverse event and the family members do not understand, medical disputes are more likely to occur, and nurses are more likely to be affected by the event, prone to frustration, and even more serious psychological and physical distress. It is suggested that operating room nursing managers should pay attention to the process of adverse events timely and accurately, intervene in time for nurses involved in the Second Victim, reduce their psychological pressure, and set up psychological counseling corner when necessary to provide positive guidance to nurses, so that they can get more management support and reduce their psychological distress.

The results of this study showed that nurses with higher career resilience were less affected by adverse events as the Second Victims. Career resilience belongs to the category of positive psychology, which can not only help employees flexibilities to adapt to the career environment and cope with challenges, but also promote the rebound or recovery of employees in the face of career difficulties (24). As a special specialty department, intraoperative nursing cooperation in operating room is increasingly updated with the changes of surgical methods and equipment. Operating room nurses need to constantly improve their ability to cope with higher requirements of work. Nurses with stronger career resilience can better face work adversity, maintain a better state of mind to deal with adverse events, and they will be less affected. In this regard, managers should formulate different training plans and corresponding stage goals according to different levels of nurses, so that nurses can improve the corresponding work skills at different work stages, enhance the work resilience of nurses, so that they can carry out corresponding measures and solutions when adverse events occur, and better promote their career development.

In addition, we found that operating room nurses with higher organizational support were less affected by adverse events as the Second Victims. When adverse events occur, patients and their families are the “first victims”, and the operating room nurses as the Second Victims also suffer from physical and mental trauma, tension, anxiety and other psychological emotions. At this time, they not only need to face the difficulties that may come from their families, but also fear the handling of hospital management such as head nurses and directors. Therefore, some nurses show an evaded attitude towards adverse events and do not take the initiative to report to managers (25). Nursing managers should pay attention to the daily work of nurses in time, correctly guide the second victims of adverse events, understand the methods of nurses to deal with adverse events, establish the discussion and analysis system of adverse events, and improve the management system of adverse events.

## 5 Limitation

The present study had certain limitations. Firstly, the subjects were local operating room limited to a province in China, and most of them were young female nurses. Therefore, the results have limited generalizability, and it is necessary to expand the number of samples to more areas and more nurses. Secondly, the cross-sectional design of the study was limited to exploring rather than causality. In the future, longitudinal studies can be conducted for further exploration.

## 6 Conclusion

The Second Victim experience of operating room nurses is mostly at a medium and high level. Responsibility in adverse events, career resilience and organizational support have a significant impact on the second victim experience level of operating room nurses. At the management level, crisis support and external help for the second victim should be provided, a support system based on the hospital or department level should be established, adverse events should be reported in a non-punitive manner, different training programs should be formulated for nurses at different levels, professional and psychological support should be provided for nurses, and the professional identity of operating room nurses should be enhanced at work. So as to further improve the level of operating room nursing and promote the construction of clinical nursing services.

## Data Availability

The original contributions presented in the study are included in the article/Supplementary Material, further inquiries can be directed to the corresponding author.
